# Supplementary material for: The partitioning of fatty acids between membrane and storage lipids controls ER membrane expansion
Source: bioRxiv. 2024 Sep 5:2024.09.05.611378. Preprint. [Version 1] doi: 10.1101/2024.09.05.611378 (PMC11398528; doi:10.1101/2024.09.05.611378)
Supplement: 1 [file NIHPP2024.09.05.611378v1-supplement-1.pdf]

bioRxiv preprint doi: https://doi.org/10.1101/2024.09.05.611378; this version posted September 5, 2024. The copyright holder for this preprint (which was not certified by peer review) is the author/funder, who has granted bioRxiv a license to display the preprint in perpetuity. It is made available under aCC-BY 4.0 International license.

Supplementary figure legends

Figure EV1. Multiple sequence alignment (Clustal Omega) of the predicted transmembrane domains of the fungal members of the PDAT family (Barbosa *et al.*, 2019). The UniProt accession numbers are included in the description on the left. Arrows indicate the position of the conserved GxxxG motif.

Figure EV2. Quantification of the BODIPY 493/503 labelling of 4Δ cells carrying the denoted plasmids; data are means ± SD from three experiments with at least 75 cells measured per strain per experiment. Statistical analysis was performed by one way ANOVA with Šidák correction; *** $p<0.001$, ****$p<0.0001$.

Figure EV3. Cells co-expressing a chromosomally integrated Faa4-mNG, Sec63-mCh and Lro1 or Lro1* from a centromeric vector, were stained with monodansylpentane (MDH) to visualize LDs.

Figure EV4. Western blots of protein extracts from 4Δ cells expressing the indicated Lro1-Protein A fusions from centromeric vectors cultured in the exponential (EXP) or post-diauxic shift (PDS) phases. Lower panels show the corresponding commassie stained gels.

Figure EV5. Wild-type cells (BY4741) carrying the indicated constructs were grown in galactose as in Fig. 2A and processed for lipidomics analysis as described under Methods. Top panel: analysis of the major PE species; bottom panel: analysis of the major PI species.

Figure EV6. *lro1*Δ cells expressing Nsr1-GFP and Lro1- or Lro1*-mCh were visualized at the EXP or PDS phases of growth. Scale bar, 5μm.

Figure EV7. Wild-type cells carrying the denoted plasmids, were grown and imaged as described in Fig. 6B; at the end-point of the assay (2 hours copper), cells were lysed and the extracts were analyzed by western blot using anti-Dgk1 antibodies. The star indicates a non-specific band. Note that the antibody does not detect the endogenous Dgk1 protein.

Figure EV8. Time lapse imaging of cells grown as detailed in Fig. 6B, to express sequentially Gal-Dgk1 and then Cup1-Lro1*. The sequence starts after cells were growing for 50 min in glucose/copper sulphate-containing media, corresponding to time point 0 of the time-lapse. Individual frames and the time points (in min) when they were captured are shown. Scale bar, 5μm.

bioRxiv preprint doi: https://doi.org/10.1101/2024.09.05.611378; this version posted September 5, 2024. The copyright holder for this preprint (which was not certified by peer review) is the author/funder, who has granted bioRxiv a license to display the preprint in perpetuity. It is made available under aCC-BY 4.0 International license.

Figure EV9. Sequential optical sections obtained by super resolution microscopy as described in Methods; cells shown are the same as the those in Fig. 7F. Scale bar, 3μm.

bioRxiv preprint doi: https://doi.org/10.1101/2024.09.05.611378; this version posted September 5, 2024. The copyright holder for this preprint (which was not certified by peer review) is the author/funder, who has granted bioRxiv a license to display the preprint in perpetuity. It is made available under aCC-BY 4.0 International license.

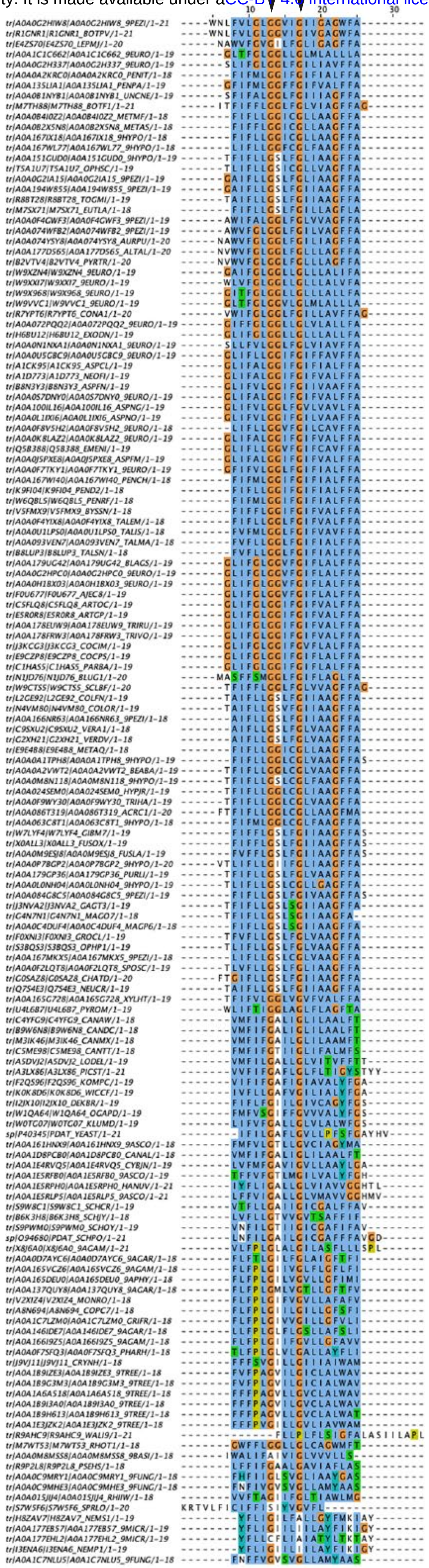

Figure EV1

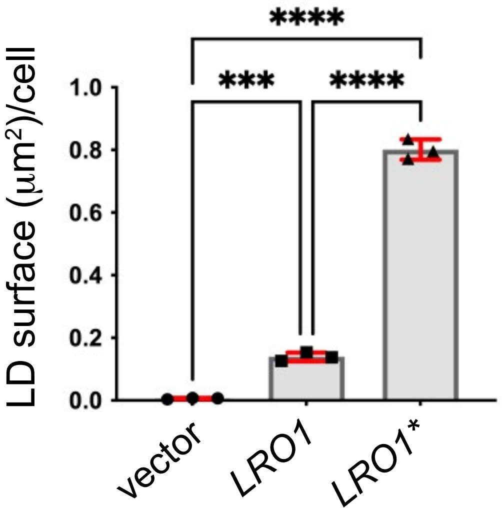

Figure EV2

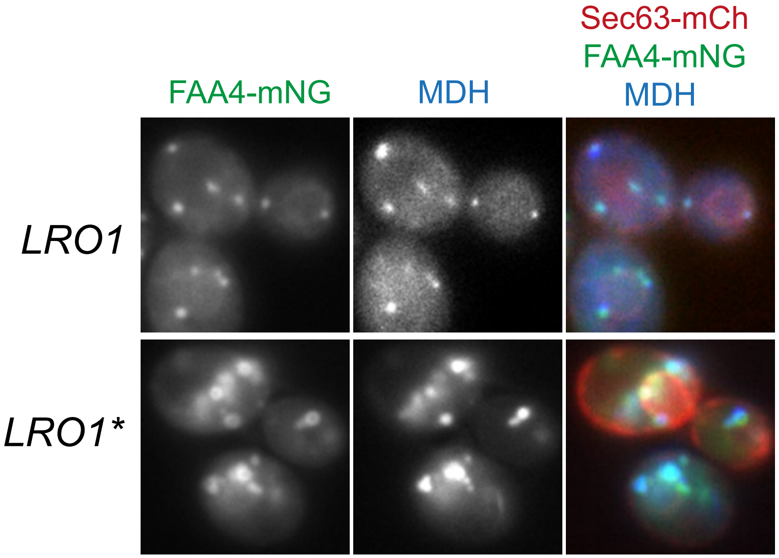

Figure EV3

bioRxiv preprint doi: https://doi.org/10.1101/2024.09.05.611378; this version posted September 5, 2024. The copyright holder for this preprint (which was not certified by peer review) is the author/funder, who has granted bioRxiv a license to display the preprint in perpetuity. It is made available under aCC-BY 4.0 International license.

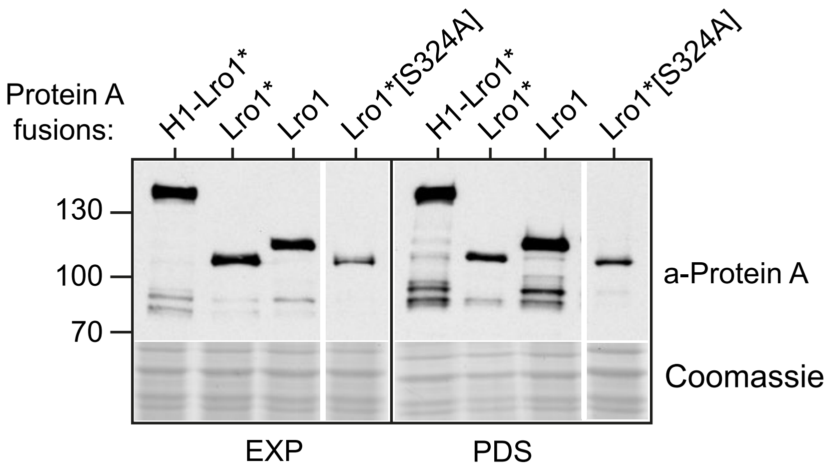

Figure EV4

bioRxiv preprint doi: https://doi.org/10.1101/2024.09.05.611378; this version posted September 5, 2024. The copyright holder for this preprint (which was not certified by peer review) is the author/funder, who has granted bioRxiv a license to display the preprint in perpetuity. It is made available under aCC-BY 4.0 International license.

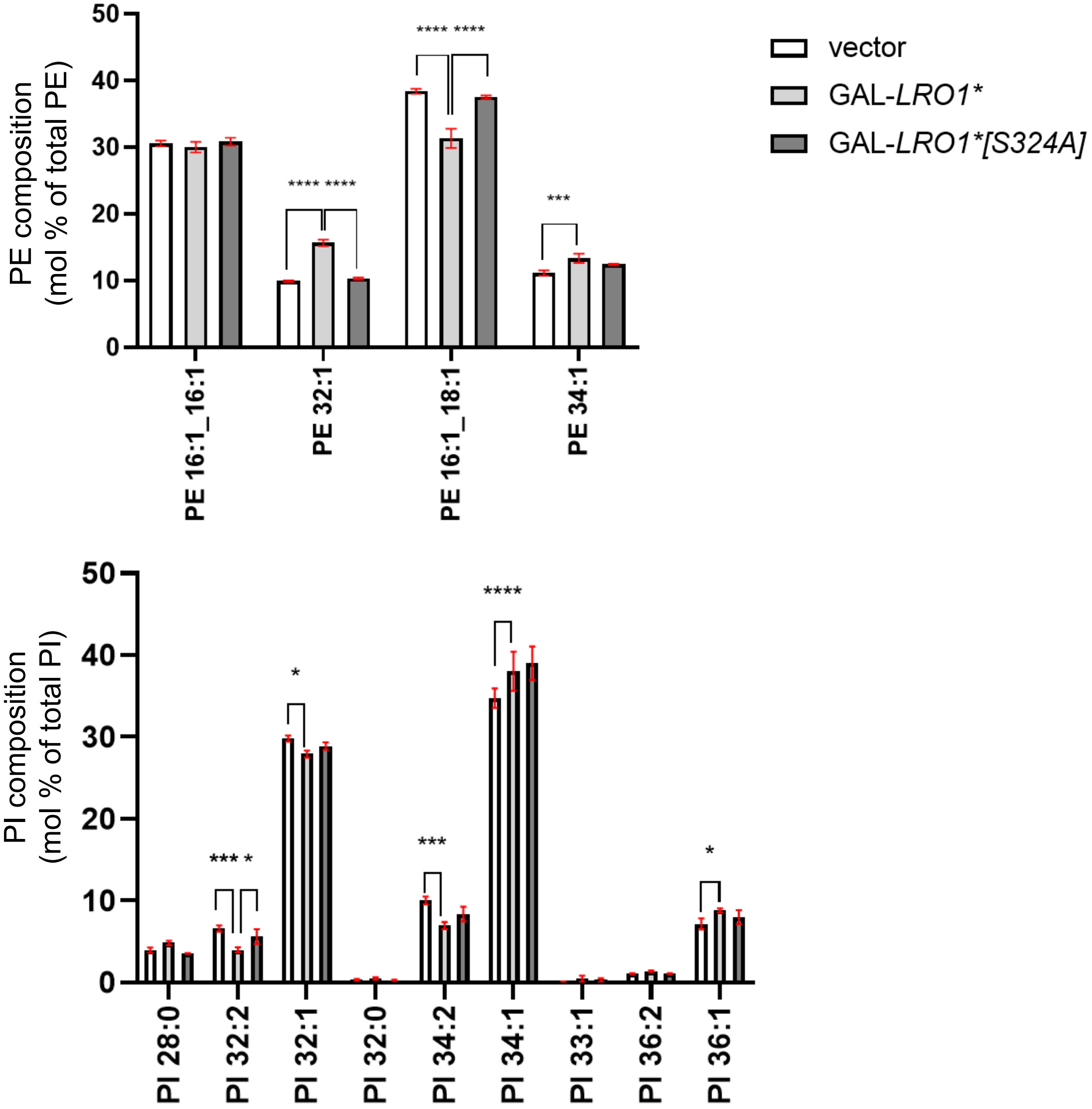

Figure EV5

bioRxiv preprint doi: https://doi.org/10.1101/2024.09.05.611378; this version posted September 5, 2024. The copyright holder for this preprint (which was not certified by peer review) is the author/funder, who has granted bioRxiv a license to display the preprint in perpetuity. It is made available under aCC-BY 4.0 International license.

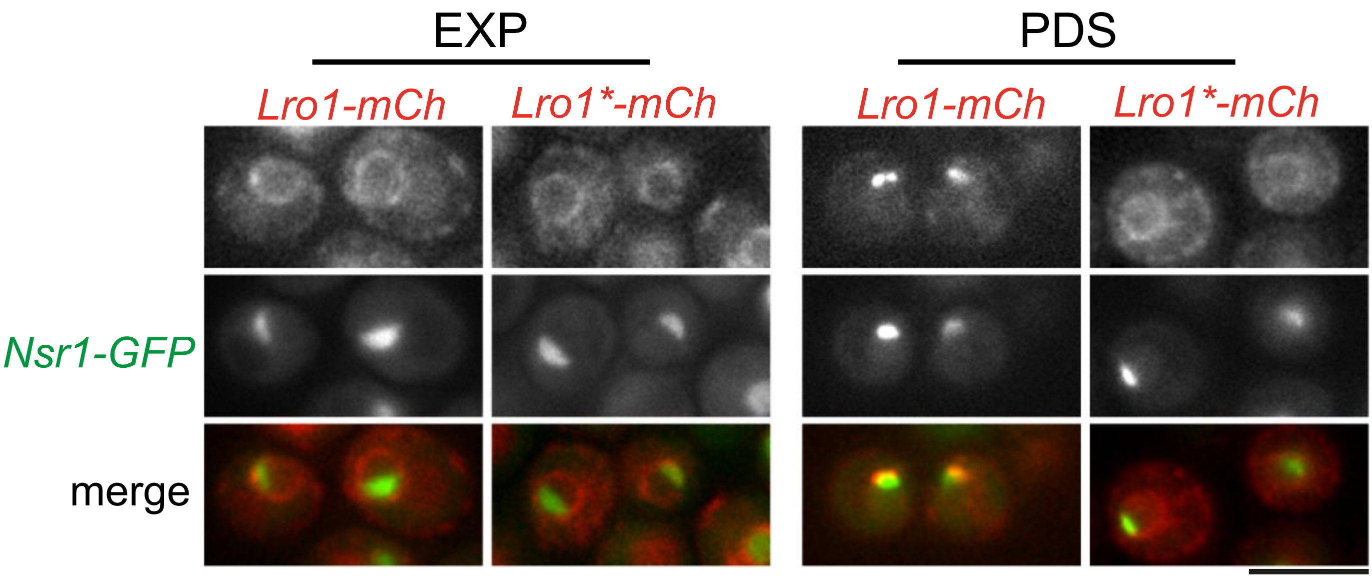

Figure EV6

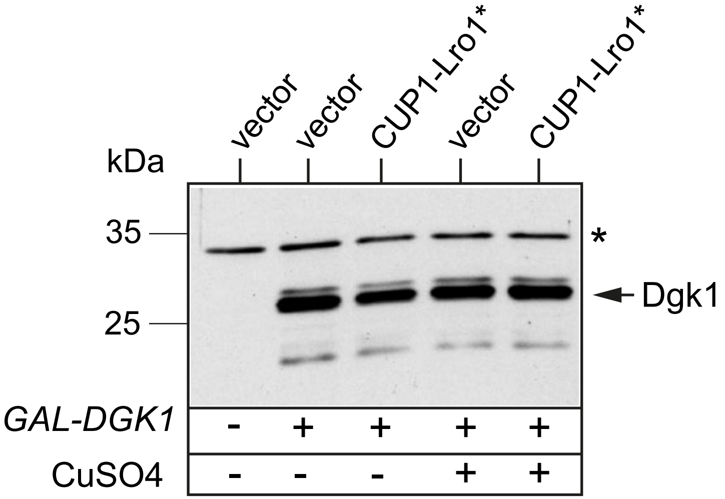

vector
vector
CUP1-Lro1*
vector
CUP1-Lro1*
kDa
35
25
*
Dgk1
GAL-DGK1
- + + + +
CuSO4
- - - + +

Figure EV7

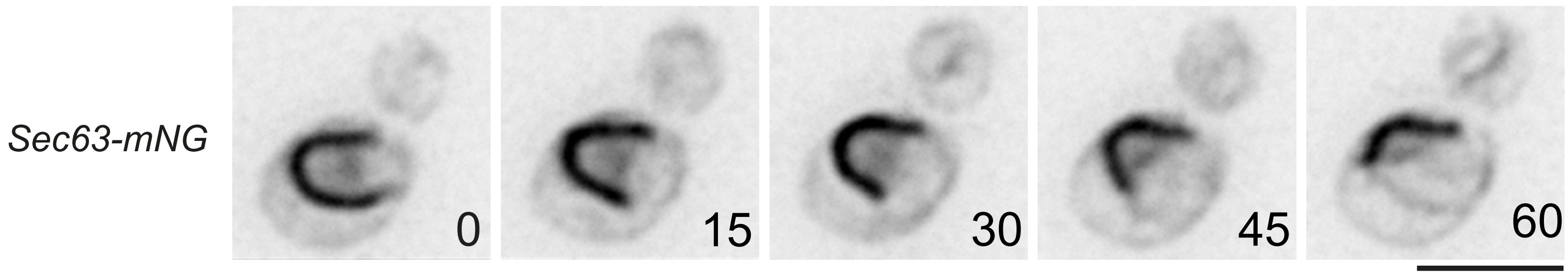

Figure EV8

LRO1/vector

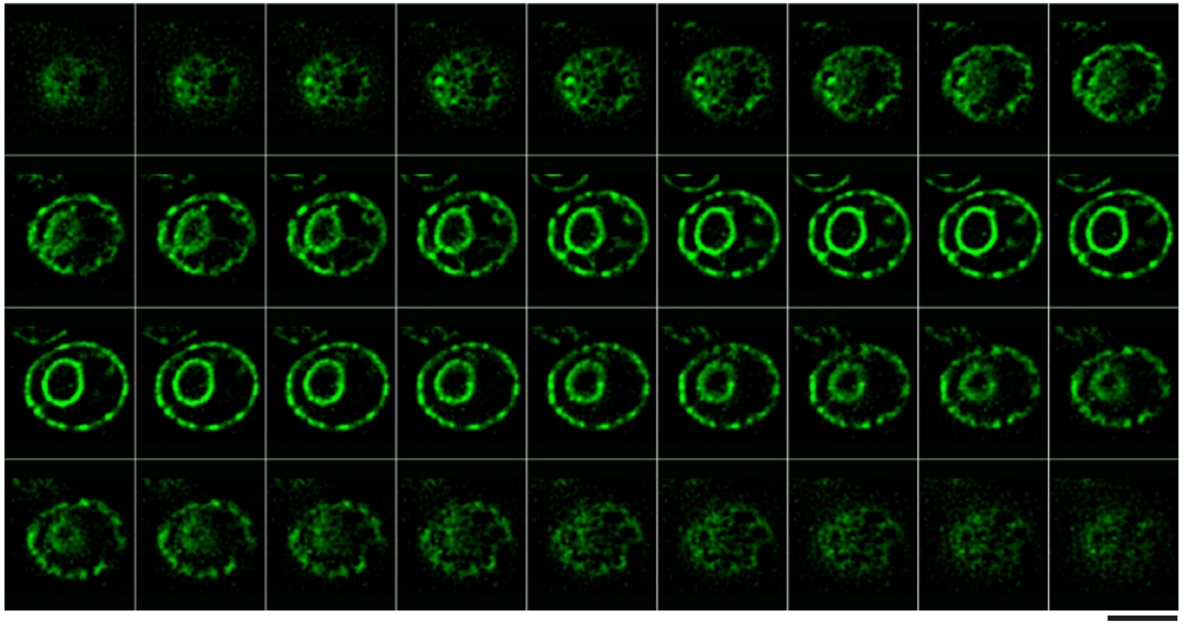

LRO1/PAH1-7A*

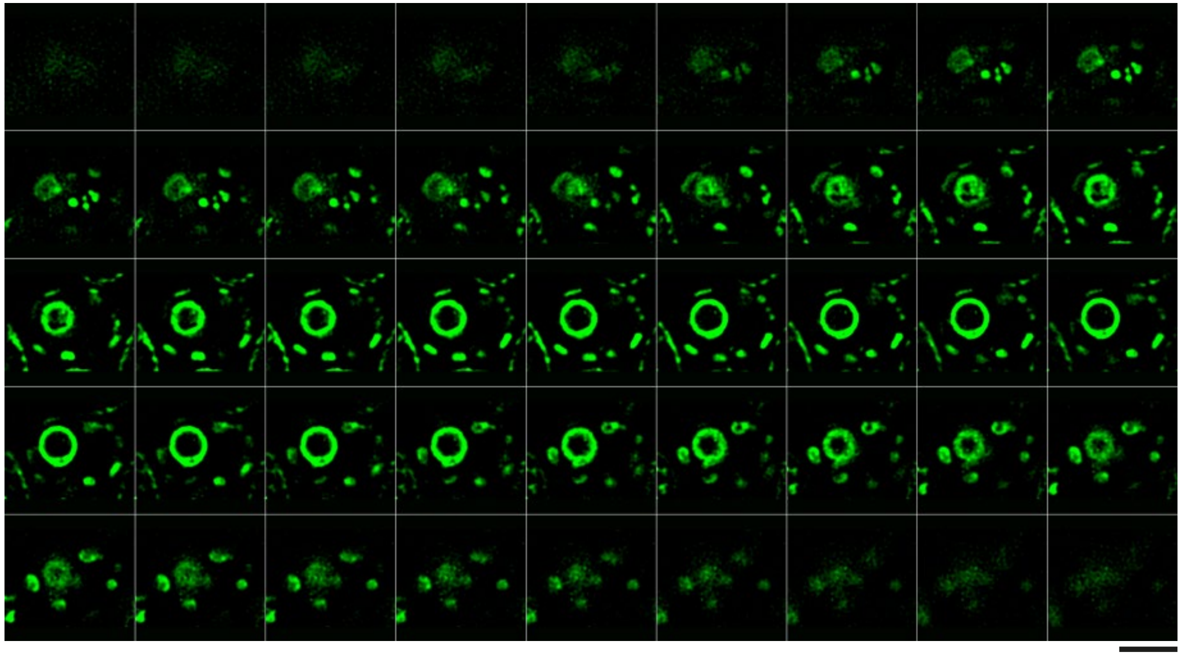

Figure EV9

bioRxiv preprint doi: https://doi.org/10.1101/2024.09.05.611378; this version posted September 5, 2024. The copyright holder for this preprint (which was not certified by peer review) is the author/funder, who has granted bioRxiv a license to display the preprint in perpetuity. It is made available under aCC-BY 4.0 International license.

**Plasmid description**	**Source/reference**	**Name**
LRO1 under control of *LRO1* promoter in *CEN/URA3* vector	Barbosa et al, 2019	YCplac33-*LRO1*
*LRO1** under control of *LRO1* promoter in *CEN/URA3* vector	This paper	YCplac33-*LRO1**
*HEH1[163-454]-LRO1** under control of *NOP1* promoter in *CEN/URA3* vector	This paper	YCplac33-*H1-LRO1**
LRO1[S324A]* under control of *LRO1* promoter in *CEN/URA3* vector	This paper	YCplac33-*LRO1*[S324A]*
LRO1[GS string] under control of *LRO1* promoter in *CEN/URA3* vector	This paper	YCplac33-*LRO1*[GS string]*
LRO1-mCherry under control of *LRO1* promoter in *CEN/URA3* vector	Barbosa et al, 2019	YCplac33-*LRO1-mCh*
LRO1-mCherry* under control of *LRO1* promoter in *CEN/URA3* vector	This paper	YCplac33-*LRO1*-mCh*
HEH1[163-454]-LRO1-mCherry* under control of *NOP1* promoter in *CEN/URA3* vector	This paper	YCplac33-*H1-LRO1*-mCh*
LRO1[S324A]-mCherry* under control of *LRO1* promoter in *CEN/URA3* vector	This paper	YCplac33-*LRO1*[S324A]-mCh*
LRO1[GS string]-mCherry under control of *LRO1* promoter in *CEN/URA3* vector	This paper	YCplac33-*LRO1*[GS string]-mCh*
LRO1-Protein A under control of *LRO1* promoter in *CEN/URA3* vector	This paper	YCplac33-*LRO1-PtA*
LRO1-Protein A* under control of *LRO1* promoter in *CEN/URA3* vector	This paper	YCplac33-*LRO1*-PtA*
HEH1[163-454]-LRO1-Protein A* under control of *NOP1* promoter in *CEN/URA3* vector	This paper	YCplac33-*H1-LRO1*-PtA*
LRO1[S324A]-Protein A* under control of *LRO1* promoter in *CEN/URA3* vector	This paper	YCplac33-*LRO1*[S324A]-PtA*
LRO1 under control of *GAL1/10* promoter in *2μ/LEU2* vector	Barbosa et al, 2019	YEplac181-*GAL1/10-LRO1*
LRO1[S324A] under control of *GAL1/10* promoter in *2μ/LEU2* vector	Barbosa et al, 2019	YEplac181-*GAL1/10-LRO1[S324A]*
*LRO1** under control of *GAL1/10* promoter in *2μ/LEU2* vector	This paper	YEplac181-*GAL1/10-LRO1**
LRO1[S324A]* under control of *GAL1/10* promoter in *2μ/LEU2* vector	This paper	YEplac181-*GAL1/10-LRO1*[S324A]*
*LRO1** under control of *CUP1* promoter in *2μ/URA3* vector	This paper	YEplac195-*CUP1-LRO1**
*LRO1** under control of *CUP1* promoter in *CEN/URA3 vector*	This paper	YCplac33-*CUP1-LRO1**
LRO1[S324A]* under control of *CUP1* promoter in *CEN/URA3 vector*	This paper	YCplac33-*CUP1-LRO1*[S324A]*
*LRO1** under control of *CUP1* promoter in *CEN/LEU2 vector*	This paper	YCplac111-*CUP1-LRO1**
PAH1-7A under control of *GAL1/10* promoter in *2μ/LEU2* vector	O'Hara et al, 2006	YEplac181-*GAL1/10-PAH1-7A*
DGK1 under control of *GAL1/10* promoter in *2μ/LEU2* vector	Han et al, 2008	YEplac181-*GAL1/10-DGK1*
DGK1 under control of *GAL1/10* promoter in *2μ/URA3* vector	Karanasios et al, 2010	YEplac195-*GAL1/10-DGK1*

bioRxiv preprint doi: https://doi.org/10.1101/2024.09.05.611378; this version posted September 5, 2024. The copyright holder for this preprint (which was not certified by peer review) is the author/funder, who has granted bioRxiv a license to display the preprint in perpetuity. It is made available under aCC-BY 4.0 International license.

PAH1-GFP under control of *PAH1* promoter in *CEN/LEU2 vector*	Karanasios et al, 2010	YCplac111-*PAH1-GFP*
PSR1[1-28]-mCherry under the control of PSR1 promoter in integrative/*HIS3* vector	This study	pRS403-*PSR1[1-28]-mCh*
LRO1-mNeonGreen* under control of *LRO1* promoter in *CEN/LEU2 vector*	Karanasios et al, 2010	YCplac111-*LRO1*-mNG*

Table S1
Plasmids used in this study.

bioRxiv preprint doi: https://doi.org/10.1101/2024.09.05.611378; this version posted September 5, 2024. The copyright holder for this preprint (which was not certified by peer review) is the author/funder, who has granted bioRxiv a license to display the preprint in perpetuity. It is made available under aCC-BY 4.0 International license.

**Genotype**	**Source/reference**	**Strain**
MATa his3Δ1 leu2Δ0 met15Δ0 ura3Δ0	Open Biosystems	BY4741
MATα his3Δ1 leu2Δ0 lys2Δ0 ura3Δ0	Open Biosystems	BY4742
MATα his3Δ1 leu2Δ0 lys2Δ0 ura3Δ0 met15Δ0 are1::KanMX are2::KanMX trp1::URA lro1::TRP dga1::Lox-HIS-Lox	Jacquier et al., 2011	RSY3077 (a.k.a. 4Δ)
BY4741 *FAA4-mNG::spHIS5*	This paper	SS3361
BY4741 *lro1Δ::KanMX*	Barbosa et al., 2019	SS3006
BY4741 *lro1Δ::KanMX HIS3::pRS403-NOP1-RFP*	This paper	SS3007
BY4741 *lro1Δ::KanMX ale1Δ::HphNT1*	This paper	SS3387
BY4741 *lro1Δ::HIS3 SEC63-mNG::KanMX*	This paper	SS3430
BY4741 *SEC63-mNG::KanMX*	This paper	SS3357
BY4741 *SEC63-mNG::KanMX RTN1-mCh::HIS3*	This paper	SS3359
BY4741 *SEC63-mNG::KanMX VPH1-mCh:: HIS3MX6*	This paper	SS3448
BY4741 *atg1Δ::HphNT1*	This paper	SS2708
BY4741 *atg1Δ::HphNT1 SEC63-mCh::HIS3*	This paper	SS3251
BY4741 *SEC63-mCh::HIS3*	This paper	SS3230
BY4741 *SEC63-mNG::KanMX HIS3::pRS403-PSR1[1-28]-mCh*	This paper	SS3434
BY4742 *pah1Δ::HIS3 pURA-PAH1*	This paper	SS2039
BY4742 *pah1Δ::HIS3 pURA-PAH1 SUR4-GFP::HphNT1*	This paper	SS3261
BY4742 *pah1Δ::HIS3 pURA-PAH1 NSR1-GFP::HphNT1*	This paper	SS3446
BY4742 *gpt2Δ::KanMX*	Open Biosystems	SS1236
BY4742 *sct1Δ::KanMX*	Open Biosystems	SS1237
BY4742 *slc1Δ::KanMX*	Open Biosystems	SS1239
BY4742 *tgl3Δ::KanMX tgl4Δ::KanMX tgl5Δ::KanMX*	Sepp Kohlwein	SS2866
BY4741 *atg15Δ::HphNT1*	This paper	SS3367
BY4741 *ale1Δ::HphNT1*	This paper	SS3376
BY4741 *dgk1Δ::HphNT1*	This paper	SS3044

Table S2
Yeast strains used in this study.
